# Smoking-associated changes in serum TP53 levels and molecular characterization of nicotine interaction with the MDM2–p53 complex: an integrated clinical and in silico study

**DOI:** 10.1016/j.jgeb.2026.100837

**Published:** 2026-10-19

**Authors:** Noor Kareem Khalifa, Maysoon Khalid Hussein

**Affiliations:** Collage of Chemistry, Baghdad University, Iraq

**Keywords:** Smoking, Tumor suppressor protein p53 level, Nicotine-MDM2-p53 complex, Molecular docking, Molecular dynamics simulation

## Abstract

Smoking induces cellular stress and genomic damage that may disrupt regulation of the p53 tumor-suppressor pathway. This cross-sectional study evaluated serum p53 concentrations in 154 Iraqi adults, including 92 smokers and 62 non-smoking controls, and examined the predicted interaction of nicotine with the MDM2–p53 complex using molecular docking and molecular dynamics simulations. Serum p53 was measured by sandwich ELISA, while smokers were classified as Cigarette, narghile, or Electronic-Cigarette users, and associations between cumulative smoking exposure and serum p53 were assessed. Nicotine was docked into the MDM2–p53 peptide complex structure (PDB ID: 4HFZ) using MOE 2019, followed by a 110-ns molecular dynamics simulation in Desmond. Serum p53 concentrations were significantly higher in smokers than in controls (4791.2 vs. 3024.1 pg/mL, *p* < 0.001). No significant differences were detected among smoking types (*p* = 0.547), and cumulative exposure was not significantly correlated with serum p53 in any subgroup. Docking identified a preferred nicotine-binding pose with a predicted score of −5.31 kcal/mol and interactions involving Val28 and Arg29. The simulated complex exhibited marked early conformational adjustment, followed by a comparatively stable trajectory supported by persistent and intermittent noncovalent interactions. These findings indicate that smoking is associated with elevated circulating p53 concentrations and suggest that nicotine may interact with residues within the MDM2–p53 binding region; however, further cellular and biochemical validation is required.

## 1 Introduction

The *TP53* gene lies on the short arm of chromosome 17 (17p13) and encodes the Tumor suppressor protein p53, which is one of the most important proteins in maintaining cell health. *TP53* is one of the most commonly mutated genes in human tumors because it plays a key role in preventing malignant transformation. Mutations in the *TP53* gene are among the most frequent genetic alterations in lung cancer; however, their prevalence varies considerably across different histological subtypes and patient demographics.^1^

One of the most well-studied Tumor suppressor proteins is p53, which is encoded by the *TP53* gene) presents critical barriers to cancer in the cell. It functions as a transcription factor that is activated upon exposure to various stresses, e.g., DNA damage resulting from damage or replication errors, and activated oncogenes. Through these actions, p53 helps prevent extensive damage or promotes cellular senescence, in which cells become permanently growth-arrested. This is why it is often called the “guardian of the genome.” When p53 is activated, it can stop the cell cycle to allow DNA repair, induce apoptosis if the damage is severe enough, or trigger cellular senescence, which halts growth permanently.^2^, ^3^

Murine Double Minute 2 (MDM2) strictly regulates p53 activity under physiological conditions, serving as a key negative regulator that maintains low p53 levels in unstressed cells. This suppression prevents premature cell cycle arrest or unwarranted apoptosis. Mechanistically, MDM2 directly binds to the N-terminal transactivation domain of p53, thereby inhibiting its transcriptional activity and promoting its degradation via the ubiquitin-proteasome system. Consequently, p53 and MDM2 form a tightly controlled negative feedback loop that is essential for maintaining cellular homeostasis.^4^, ^5^

The p53 protein is a transcription factor that binds to particular DNA response regions and controls the expression of several target genes. In addition to its traditional functions in cell-cycle arrest and death, p53 is implicated in stem cell control, cellular senescence, energy metabolism, oxidative stress responses, and the preservation of genomic stability.^6^

Because *TP53* mutations are common in many cancers, therapeutic strategies have been developed to restore mutant p53 function. Small molecules known as p53 reactivators can bind to mutant p53 protein and partially restore its wild-type conformation and tumor-suppressive activity.^7^, ^8^

Smoking involves the combustion of a material, predominantly tobacco, allowing the smoke to be tasted or inhaled; it can harm every aspect of the body. The predominant technique of smoking is cigarette consumption; however, alternative options encompass pipes, cigars, bidis, narghile (waterpipe), vaporizers, and bongs. Tobacco, a plant in the genus Nicotiana within the family Solanaceae, has a multifaceted character.^9^

Despite smokers frequently claiming sensations of relaxation, heightened alertness, or tranquility following nicotine consumption, tobacco smoke comprises numerous detrimental chemicals. This includes polycyclic aromatic hydrocarbons, heavy metals such as cadmium, nickel, and arsenic, and several other hazardous and carcinogenic substances. These factors collectively induce oxidative stress, inflammation, and the onset of cancer.^10^

Narghile, also known as argileh, shisha, hookah, hubble-bubble, and waterpipe, is a device employed for the inhalation of tobacco through water filtration. Contrary to prevalent perceptions, narghil smoking is not a safe substitute for cigarette smoking and is linked to numerous detrimental health impacts. Research assessing the impact of smoking on biochemical parameters has indicated changes in total protein levels and alpha-amylase activity in the sera of Iraqi narghile smokers, implying that tobacco exposure may affect systemic metabolic and inflammatory responses.^11^

Cigarette smoking (CK) represents a major source of genotoxic stress, as it exposes cells to a wide range of carcinogenic compounds, particularly affecting the respiratory epithelium. Under normal circumstances, DNA damage induced by these agents activates wild-type p53, leading to cell-cycle arrest or apoptosis, thereby preventing the survival of damaged cells.^12^ Prolonged exposure to cigarette smoke can progressively diminish this protective reaction. When p53 activity is diminished due to genetic variation, disruption of the MDM2–p53 regulatory pathway, or substantial DNA damage, impaired cells may persist in growth and division.^13^

Tobacco smoke is known to impact the *TP53* gene, potentially leading to dysfunction. These alterations frequently disrupt the normal function of p53 or produce mutant p53 proteins that induce cancer. Such alterations are frequently observed in tumors associated with smoking, particularly lung cancer.^12^, ^14^ Generally, smoking exerts significant stress on the body. Various methods of regulating the MDM2–p53 axis may influence an individual's susceptibility to cancer. Disrupting this exquisitely Balanced contact is a crucial factor in the process of cancer development associated with tobacco.^13^

Nicotine is the primary addictive component of cigarette smoke; yet, it is not directly mutagenic. It has been shown to influence various cellular pathways associated with oxidative stress, inflammation, and tumor proliferation. Modulations in p53 expression have been observed in smokers; however, the underlying molecular mechanisms, particularly the potential direct influence of nicotine on the p53-MDM2 interaction, remain insufficiently elucidated.^14^

The present study integrates biochemical assessment of serum p53 levels in smokers compared with controls*,* in silico molecular docking, and molecular dynamics simulations to investigate the potential influence of nicotine on the MDM2-p53 regulatory axis. This study seeks to elucidate the impact of smoking on p53 regulation and its role in disease formation by integrating clinical data with computational techniques.

## 2 Materials and methods

### 2.1 Study population

A cross-sectional study included 154 Iraqi volunteers, aged between 26 and 64 years, comprising 92 smokers and 62 healthy non-smokers. The smoking cohort was further stratified based on the route of tobacco consumption: Cigarette smokers (*n* = 50), narghil smokers (*n* = 24), and Vape users (*n* = 18). Participants were prospectively recruited at Al-Yarmouk Teaching Hospital and Baghdad Teaching Hospital between November 2024 and March 2025, in accordance with strict inclusion criteria.

The study included Exclusion criteria such as rheumatoid arthritis, other inflammatory disorders, and persistent illnesses. Diseases such as diabetes mellitus, cardiovascular illness, liver cirrhosis, malignant tumors, or the utilization of drugs or supplements not aligned with the study protocol.

There were no significant differences between smokers and controls in terms of age or body mass index (BMI) (*p* > 0.05). Venous blood samples (10 mL) were collected from each participant in gel vacutainer tubes. Samples were centrifuged at 3000 rpm for 10 min to obtain serum, which was transferred to Eppendorf tubes and kept at −20 °C until analysis. The study was approved by the Ethics Committee of the College of Science, University of Baghdad (Ref. CSEC/0325/0054). Written informed consent was obtained from all participants before inclusion in the study.

### 2.2 Smoking history and cumulative tobacco exposure assessment

Smoking history was obtained from all participants using a structured questionnaire that recorded the type of smoking product, smoking frequency, duration of use, and age at smoking initiation. Cumulative tobacco and nicotine exposure was expressed as pack-years for a conventional cigarette smoker and equivalent cumulative exposure indices for narghil and electronic cigarette users. For conventional cigarette smokers, pack-years were calculated using the standard Eq..^15^

**Pack-Years = (number cigarettes smoked per Day ÷ 20) × Years of smoking.**

*Where 20 cigarettes represent one standard cigarette pack.

For narghil smokers, cumulative exposure was estimated based on the average number of smoking sessions per day and the duration of smoking. Previous studies have estimated that a single narghil session may deliver smoke exposure equivalent to approximately 19 Cigarettes during a 15-min session and up to 225 cigarettes during a 3-h session, depending on smoking duration and puffing behavior.^16^ Accordingly, cumulative narghil exposure was calculated by multiplying the average number of daily smoking sessions by the total duration of narghil use (years).

For electronic cigarette (vaping) users, cumulative exposure (vape–years) was estimated according to a previously validated approach. The reported number of puffs per day was divided by 10, assuming that approximately 10 puffs are equivalent to smoking one conventional cigarette. This value was adjusted based on the number of vaping days per month (divided by 30 to obtain an average daily use). The resulting cigarette–equivalent value was then divided by 20 to convert it to pack equivalents, and multiplied by the duration of vaping (in years), as shown below.^17^

**Vape-Years = ((Puffs/day) / 10) × ((Days vaped/month) / 30) × 1 /20 ×** **Years of vaping.**

To facilitate comparisons among conventional cigarette smokers, narghil smokers, and electronic cigarette users, cumulative nicotine exposure was expressed as cigarette-equivalent pack-years whenever feasible. Because patterns of tobacco and nicotine consumption differ substantially across smoking modalities, published conversion factors were used to estimate cigarette-equivalent exposure. Although these conversion methods provide an approximate measure rather than a direct biological equivalent, they offer a practical and standardized approach for comparing cumulative exposure among different smoking products and have been adopted in previous epidemiological studies.

These cumulative exposure indices were used to investigate the association between long-term tobacco or nicotine exposure and the measured biochemical parameters.

The estimation of cumulative smoking exposure for electronic cigarette and narghil users was based on currently available approximation methods. Because no universally accepted method currently exists for quantifying cumulative exposure among narghil and electronic cigarette users, the pack-year calculations used in this study should be considered approximate estimates rather than standardized measures. These approaches were adopted to provide a consistent assessment of cumulative tobacco and nicotine exposure across different smoking modalities.

### 2.3 Measurement of human tumor protein (p53) levels

Serum p53 concentrations were quantified employing a commercially available sandwich ELISA kit (Elabscience, Wuhan, China; catalog No. *E*-EL-H0910), adhering to the manufacturer's specified protocol. Absorbance was then assessed at 450 nm utilizing a Huma Reader HS microplate reader (Human Diagnostics Worldwide, Germany). Concentrations were calculated from a standard calibration curve and reported in pg**/**ml.

### 2.4 Statistical analysis

Statistical analyses were performed using the Statistical Package for the Social Sciences (SPSS) version 26.0 (IBM Corp., Armonk, NY, USA). Continuous variables were expressed as mean ± standard deviation (SD), whereas categorical variables (such as sex distribution) were presented as counts and percentages. The normality of data distribution was assessed using the Kolmogorov–Smirnov test, while homogeneity of variances among groups was evaluated using Levene's test. Since the data met the assumptions of normality and homogeneity of variance, parametric statistical tests were applied. Categorical variables and baseline demographic comparisons (sex distribution among groups) were analyzed using the Chi-square test. Comparisons of Continuous variables among the three study groups were performed using one-way analysis of variance (ANOVA), followed by Tukey's Honestly Significant Difference (HSD) post hoc test for multiple comparisons. Differences between male and female participants were evaluated using the independent-samples *t*-test. Pearson's correlation coefficient was used to assess the relationships between serum p53 levels and other continuous variables. A *p*-value of less than 0.05 was considered statistically significant.

### 2.5 Molecular docking method

Molecular docking calculations were performed using MOE 2019 (Molecular Operating Environment) software suite.^18^ The crystal structure of the target protein **MDM2-p53 Peptide Complex** was retrieved from the RCSB Protein Data Bank (PDB ID: 4HFZ), as shown in Fig. 2. Protein preparation involved the removal of extraneous heteroatoms, the addition of hydrogen atoms, the correction of atom types, and the assignment of appropriate protonation states using the protonate 3D module in MOE.^19^ Protonation was performed under physiological conditions at approximately pH 7.0–7.4. The AMBER10:EHT force field was used during protein preparation and energy minimization.^20^

Rather than employing a conventional grid box. The docking region was defined around the protein's active-site pocket. The binding site was centered on the coordinates of the co-crystallized ligand within the identified active pocket of 4HFZ, thereby restricting the conformational search space to the residues that define this native binding region. Docking was performed using the triangle-matcher placement method, and the initial poses were scored using the London dG scoring function (*E*-score1). The top-scoring poses underwent energy minimization (refinement) within the binding pocket using the AMBER 10:EHT force field, and the final 5 docking poses were re-ranked using the GBVI/WSA dG free-energy scoring function (E-score 2). Furthermore, the Root-Mean-Square Deviation (RMSD) values were calculated for the generated docking poses to evaluate their conformational stability and structural deviation within the binding cavity. Prior to docking, crystallographic water molecules were carefully inspected, and non-essential waters were eliminated to prevent artificial steric hindrance.

After the optimization process, the nicotine structure was brought into MOE 2019 and stored in the ligand database for future molecular docking experiments (Fig. 1).

**Fig. 1:**
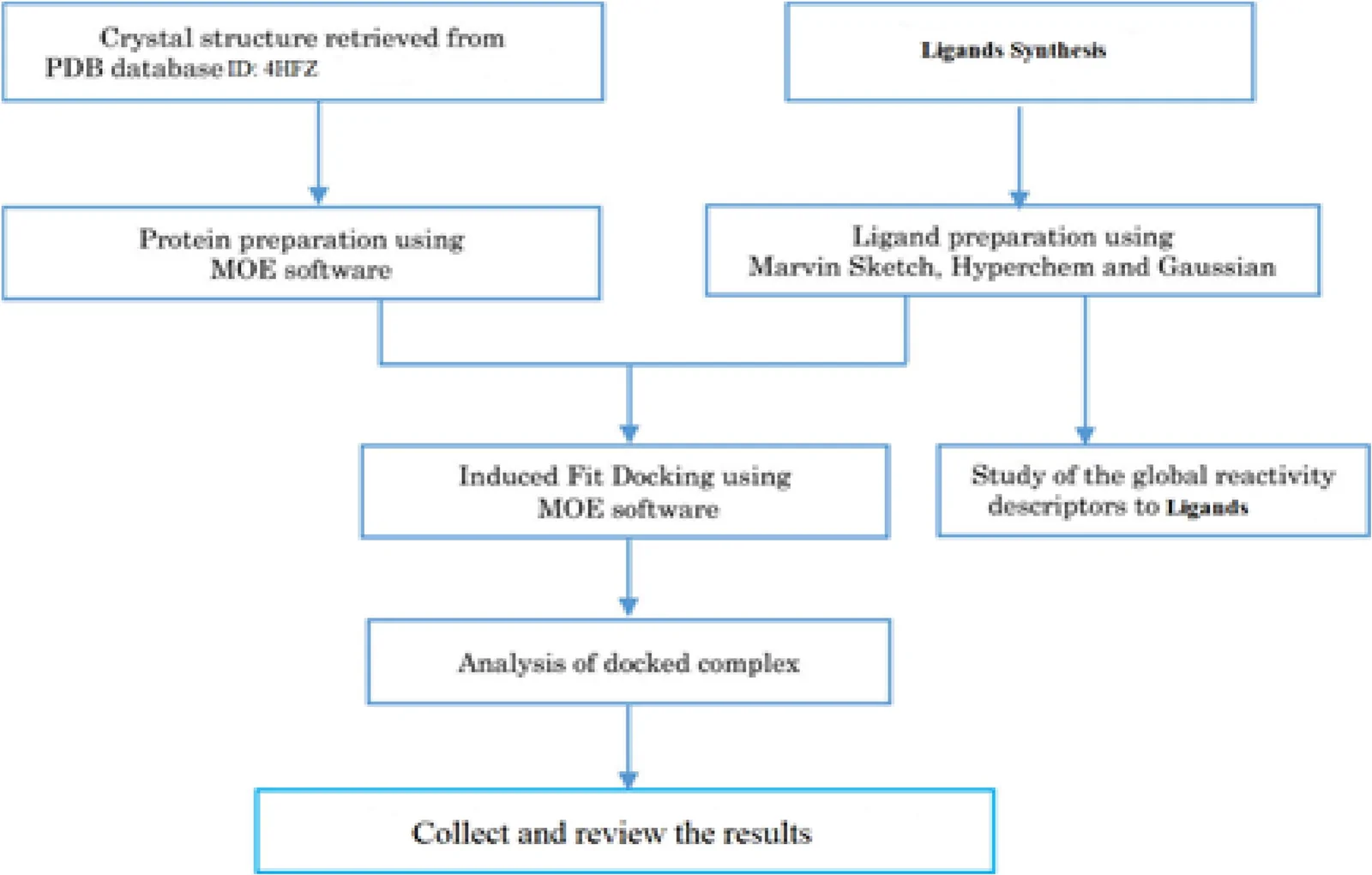
A diagram shows how docking works, how ligands are analyzed, and how they react.

The crystal structure of the protein target (PDB ID: 4HFZ) was retrieved from the Protein Data Bank at a resolution of 2.69 Å. A resolution between 1.50 and 3.00 Å is generally considered of suitable quality for molecular docking and simulation studies (Fig. 2).

**Fig. 2:**
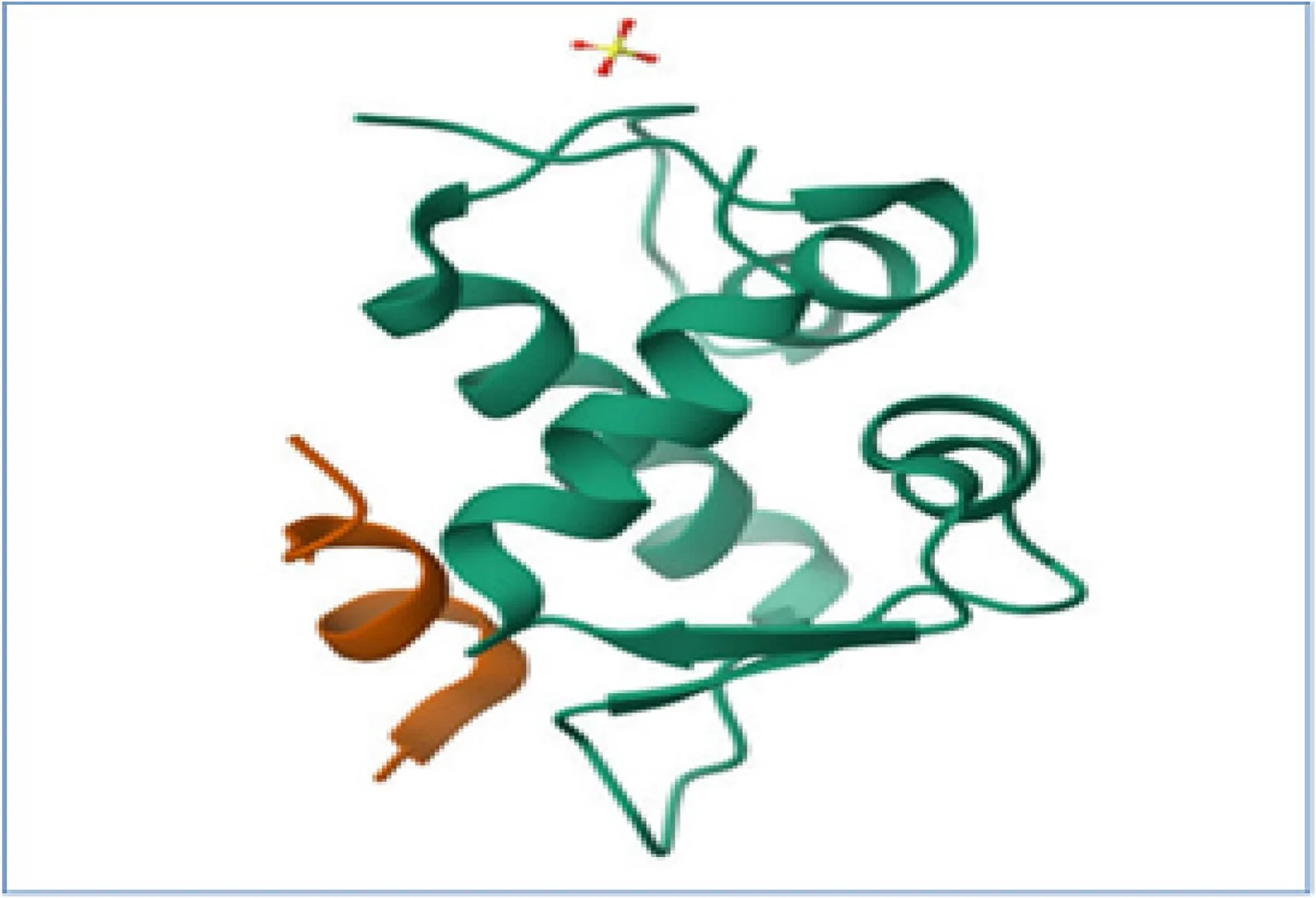
Crystal structures of the 4HFZ protein.

### 2.6 Molecular dynamics simulation methodology

Molecular dynamics (MD) simulations were conducted using the Desmond software package within the Maestro interface.^21^ Visualization, data analysis, and the generation of publication-quality figures were performed using PyMOL and OriginPro.^22^ The simulations employed established and validated computational methodologies to ensure both reliability and reproducibility. The protein–ligand complex was prepared by selecting the best docking orientation of nicotine bound to the protein and importing the coordinates into the Maestro platform.

The system was solvated within an orthorhombic simulation box filled with the TIP3P water model, maintaining a minimum buffer distance of 10 Å between the solute atoms and the box boundaries. To mimic realistic physiological conditions, the system was neutralized by adding counterions (Na^+^/Cl^−^) to achieve a standard salt concentration of 0.15 M. The OPLS4 force field was used to represent all interatomic interactions.^23^ System minimization and equilibration were executed using Desmond's standard relaxation protocol. Subsequently, a production MD simulation was run for 110 ns under an NPT ensemble (at 300 K and 1.013 bar) with a time step of 2 fs, and trajectory frames were saved every 10 ps for subsequent detailed analysis. Post-simulation trajectory analyses were conducted to evaluate the structural stability and dynamic behavior of the complex using root-mean-square deviation (RMSD), root-mean-square fluctuation (RMSF), radius of gyration (*Rg*), hydrogen-bond profiling, and non-covalent interaction analyses*.*

## 3 Results

### 3.1 Demographic characteristics

Table 1 summarizes the demographic analysis, which showed that the study groups were identical and homogeneous with respect to age (*p* = 0.074) and BMI (*p* = 0.981). However, the sex distribution showed a statistically non-significant difference between the control and smoker groups (*p* = 0.947), indicating that the groups were well-matched with respect to sex distribution.

**Table 1:** Demographic characteristics in control and smoker groups.⁎

***Variable***	**Control *n*** **=** **62**	**Smokers *n*** **=** **92**	***P -* value**
***Age (years)***	*39.31 ± 10.81*	*37.32 ± 10.30*	*0.074*
***BMI (kg/m***^***2***^***)***	*28.53 ± 5.49*	*28.37 ± 5.03*	*0.981*
**Smoking type n (%)**	–		–
*Cigarette*		50 (54.3%)	
*Narghile*		24 (26.1%)	
*Vape*		18 (19.6%)	
**Pack-years**	–		–
Cigarette		29.94 ± 13.46	
Narghile		11.07 ± 9.15	
Vape		14.90 ± 6.55	
**Sex (M/F)**	35 / 27	51/41	0.947

⁎ Data are presented as mean ± SD or number (%). Statistical significance was set at *p* < 0.05.

### 3.2 Correlation analysis

Table 2 shows that a Correlation analysis was conducted to assess the relationship between serum p53 concentration and cumulative smoking exposure (pack-years) within each smoking group. No statistically significant correlation was observed among Cigarette smokers (*r* = 0.010, *p* = 0.929), Vape users (*r* = −0.418, *p* = 0.075), or Narghile smokers (*r* = −0.019, *p* = 0.910). A moderate inverse correlation was observed in the vape group; however, this relationship did not reach statistical significance.

**Table 2:** Correlation between types of smoking and p53 level.

**Group**	***r***	***p*- value**
**Cigarette**	0.010	0.929
**Vape**	−0.418	0.075
**Narghile**	−0.019	0.910

### 3.3 Serum p53 levels

Conversely, circulating p53 levels were significantly higher in smokers relative to controls (*p* < 0.001), indicating a robust correlation between smoking status and increased p53 concentration.

Moreover, as indicated in Table 3, no statistically significant changes in p53 levels were detected among smokers when categorized by smoking type (Cigarette, Vape, or Narghile) (*p* = 0.547). (See Table 4.)

**Table 3:** Comparison of serum p53 levels between smokers and controls, stratified by smoking style and sex (in smokers).

**Parameter**	**Groups**	**Mean**	**SE**	***p*- value**
**p53 (pg/mL)**	**Control**	3024.10	295.35	<0.001
	**Smokers**	4791.20	411.21	
	**Cigarette**	4374.30	588.38	0.547
	**Narghile**	5294.28	819.95	
	**Vape**	5278.76	761.24	
	**Male**	3691.31	467.10	0.002
	**Female**	6286.04	668.89

**Table 4:** The binding affinity and RMSD results of the 4HFZ protein from the docking process.

Compound	Mseq	Binding affinity (kcal/mol)	RMSD (Å)	E-conf	E-place	E-score 1	E-refine	E-score2
Nicotine-pose 1	1	−5.31	1.94	22.62	−6.07	−6.32	−19.03	−5.31
Nicotine _pose 2	1	−5.11	1.58	21.88	−5.88	−6.57	−18.81	−5.11
Nicotine-pose3	1	−4.15	2.56	16.16	−6.14	−6.65	−16.64	−4.15
Nicotine-pose 4	1	−4.15	2.91	22.50	−6.55	−6.36	−18.00	−4.15
Nicotine-pose 5	1	−4.12	2.29	22.58	−6.64	−6.79	−18.82	−4.12

### 3.4 Docking analysis

Molecular docking showed that nicotine binds to the 4HFZ protein complex with a binding affinity of −5.31 kcal/mol. The amino acid residues Val28 and Arg29 were important in the interactions, suggesting they may help maintain the stability of the nicotine–protein complex. The affinities are between −5.31 and − 4.12 kcal/mol. The table shows energy and score phrases from docking software, like *E*-conf, E-place, E-score1, E-refine, and E-score2. The final score, E-score2, is the same as the Binding Affinity for all positions, which suggests that it is the most important measure.

Table 5 confirms that Nicotine-pose1 has the best binding affinity (−5.31 kcal/mol) and an RMSD of 1.94 Å. The interacting amino acid residues of the protein are Val28 and Arg29.

**Table 5:** Details of the best poses of ligands (Nicotine) with protein 4HFZ.

Compound	Mseq	Binding affinity (kcal/mol)	Atom of a compound	Atom Receptor	Receptor Residues	Type of interaction bond	d(Å)	E (kcal/mol)
Nicotine-pose 1	1	−5.31	N 2 N 2 N 11 6-ring	CA N NH2 NE	Val 28 Arg29 Arg29 Arg29	(A) H-acceptor (A) H-acceptor (A) H-acceptor (A) pi-cation	3.58 3.39 3.07 4.11	−0.5 −0.3 −5.5 −0.4

Symbol d = Distance.

### 3.5 Molecular docking of nicotine with 4HFZ protein

The binding interactions between nicotine and the 4HFZ protein were successfully evaluated using molecular docking simulations. As illustrated in the 2D interaction diagram and 3D binding surface (Fig. 3), Nicotine-pose1 exhibited optimal accommodation within the hydrophobic binding pocket of the target macromolecule.

**Fig. 3:**
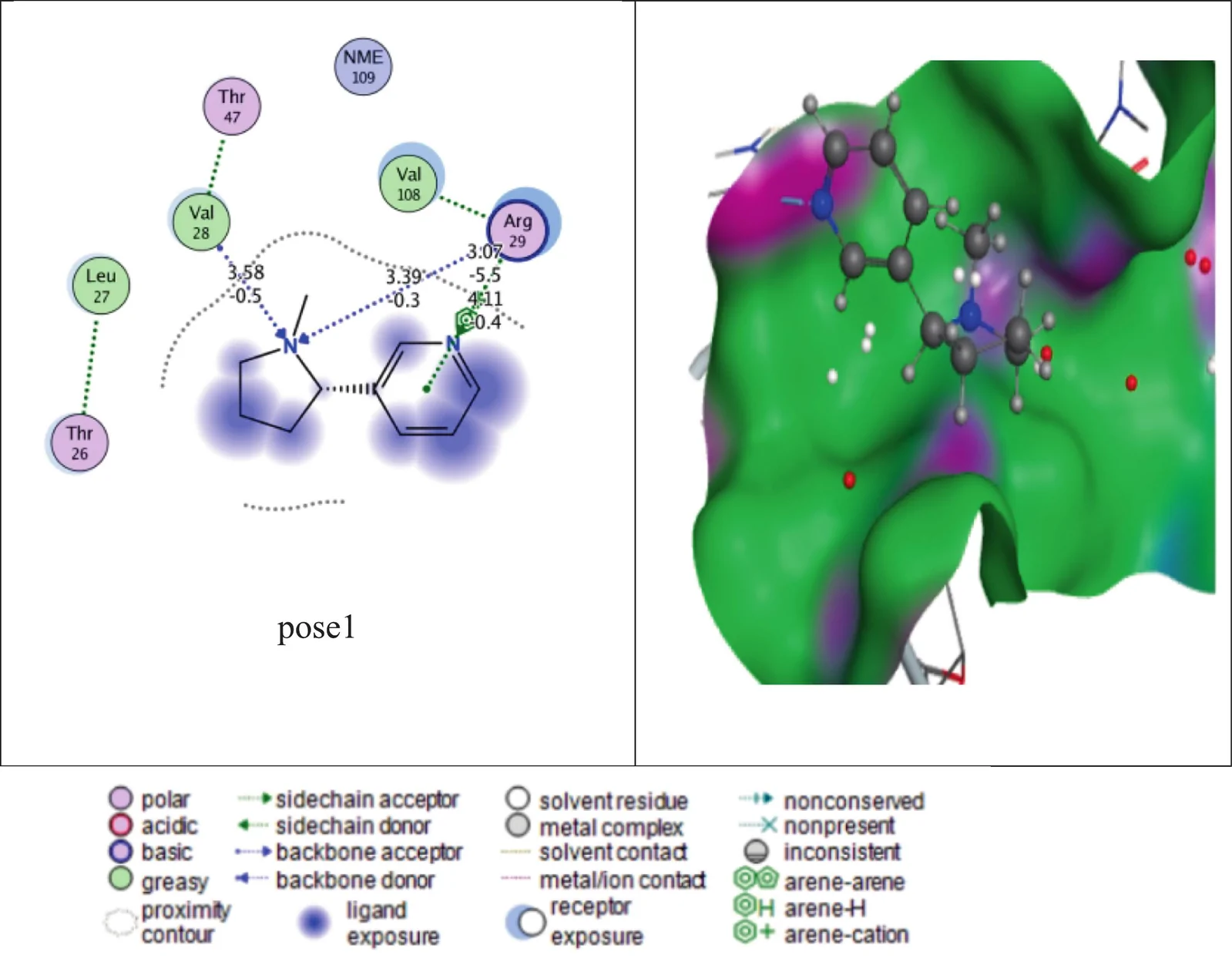
2D interactions of the inspected compounds with the key amino acid residues of the (4HFZ) protein.

The docking data demonstrated that nicotine stabilization within the active pocket is driven by a network of precise noncovalent interactions with key amino acid residues. Specifically, the nitrogen atom of the pyrrolidine ring participates in hydrogen bonding with the backbone atoms of Val 28 (d = 3.58, E = −0.5 kcal/mol) and Arg29 (d = 3.39 Å, E = −0.3 kcal/mol). Crucially, a highly favorable and robust interaction was established between the pyridine nitrogen of nicotine and the side-chain amino group of Arg29, with a close distance of 3.07 Å and a strong interaction energy of −5.5 kcal/mol. Furthermore, a π-cation interaction was observed between the aromatic ring of nicotine and Arg 29 (d = 4.11 Å, E = −0.4 kcal/mol). Additional spatial stabilization and close contacts within the cavity are provided by neighboring residues, including Thr 47, Val 108, and Leu 27, collectively locking the ligand into its native conformation without any water-mediated interference.

### 3.6 Molecular dynamics simulation

#### 3.6.1 Root mean square deviation (RMSD)

The conformational stability and structural transition of the Nicotine-4HFZ complex were evaluated over a 110 ns molecular dynamics (MD) simulation trajectory (Fig. 4). The RMSD profile exhibited a sharp initial fluctuation during the first 15 ns, reaching up to ~15.5 Å. This initial phase corresponds to a major structural equilibration and dynamic rearrangement, wherein the docking-derived pose adapts to the explicit aqueous environment and flexible loop regions undergo substantial displacement. Following this relaxation period, the system reached a distinct plateau, stabilizing at an average RMSD of ~8 Å for the remainder of the production run (20–110 ns). While an 8 Å RMSD indicates a significant global conformational shift from the initial docked starting structure, the absence of continuous drift and the persistence of a flat plateau demonstrate that the complex successfully transitioned into a stable, well-equilibrated conformational state without undergoing structural denaturation**.**

**Fig. 4:**
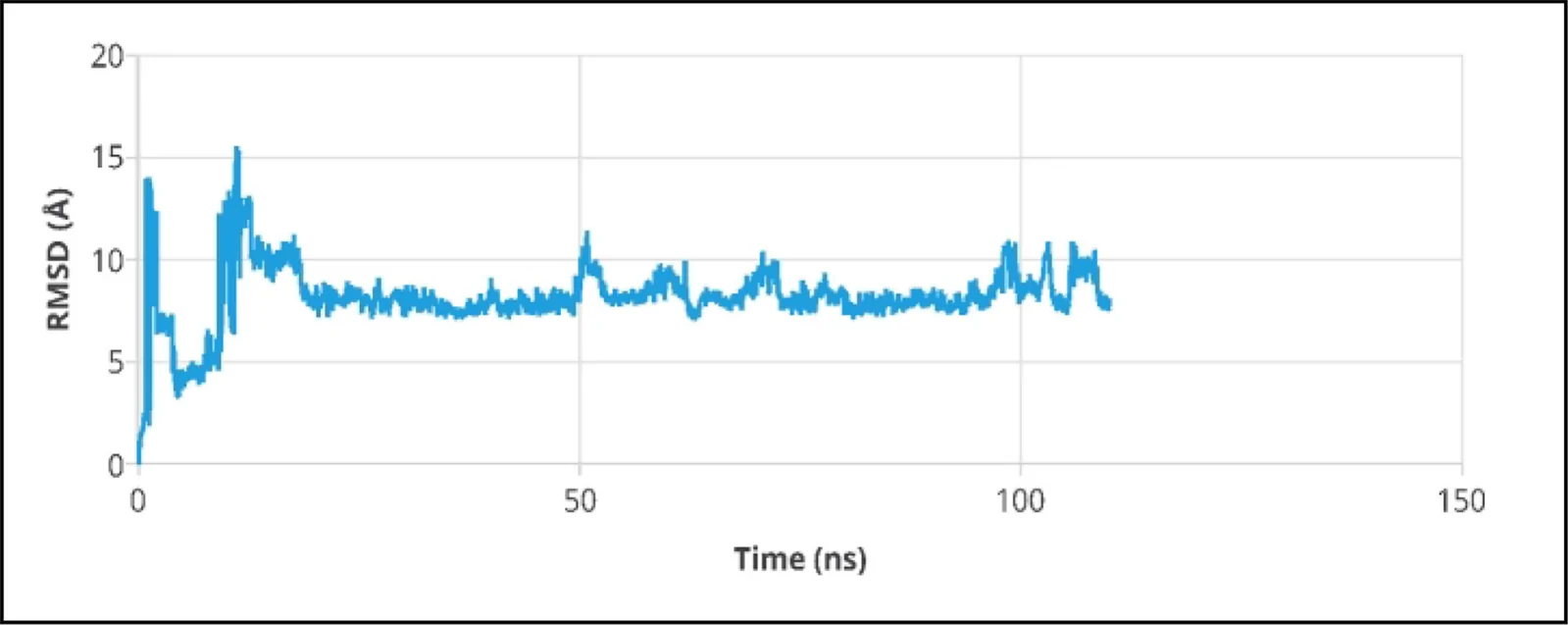
The Root Mean Square Deviation (RMSD) at 110 ns during MD.

#### 3.6.2 Non-covalent interactions

The dynamic evolution and persistence of non-covalent interactions throughout the (110 ns) simulation trajectory were evaluated quantitatively and visually. As shown in Table 6, the simulation path was analyzed across 20 discrete segments, each lasting 5.5 ns. To complement these interval-based quantitative values, a continuous, time-dependent interaction-fraction graph (Fig. 5) was used to track the continuity and frequency of hydrogen-bond, π-π stacking, and π-cation interactions over the entire trajectory. The result reveals that hydrogen bonding fluctuated significantly over time, with the highest interaction fraction recorded at 0.16 during the 33.0–38.5 ns interval and 0.14 during the 22.0–27.5 ns interval. Conversely, salt bridge interaction emerged as the most predominant and persistent interaction sustaining the complex, peaking at an interaction fraction of 0.20 between 55.0 and 60.5 ns. Interactions involving π-π stacking and π-cation were transient and predominantly confined to the early stages of the simulation (up to11.0 ns), as visually demonstrated by the initial peak in Fig. 5.

**Table 6:** Quantitative interaction fractions of hydrogen bonds, salt bridges, π-π stacking, and π-cation interactions calculated at 5.5 ns discrete time intervals during the 110 ns MD simulation**.**

**Frame**	**Time (ns)**	**Hydrogen Bonds**	**Salt Bridges**	**π-π Stacking**	**π-Cation**
26	0.0–5.5	0.02	0.02	0.13	0.08
76	5.5–11.0	0.00	0.00	0.08	0.04
126	11.0–16.5	0.04	0.08	0.00	0.00
176	16.5–22.0	0.06	0.06	0.00	0.00
226	22.0–27.5	0.14	0.02	0.00	0.00
276	27.5–33.0	0.00	0.08	0.00	0.00
326	33.0–38.5	0.16	0.04	0.00	0.00
376	38.5–44.0	0.08	0.06	0.00	0.00
426	44.0–49.5	0.06	0.06	0.00	0.00
476	49.5–55.0	0.00	0.06	0.00	0.00
526	55.0–60.5	0.02	0.20	0.00	0.00
576	60.5–66.0	0.00	0.04	0.00	0.00
626	66.0–71.5	0.02	0.06	0.00	0.00
676	71.5–77.0	0.02	0.04	0.00	0.00
726	77.0–82.5	0.04	0.04	0.00	0.00
776	82.5–88.0	0.04	0.00	0.00	0.00
826	88.0–93.5	0.00	0.02	0.00	0.00
876	93.5–99.0	0.00	0.14	0.00	0.00
926	99.0–104.5	0.00	0.14	0.00	0.00
976	104.5–110.0	0.02	0.02	0.00	0.00

**Fig. 5:**
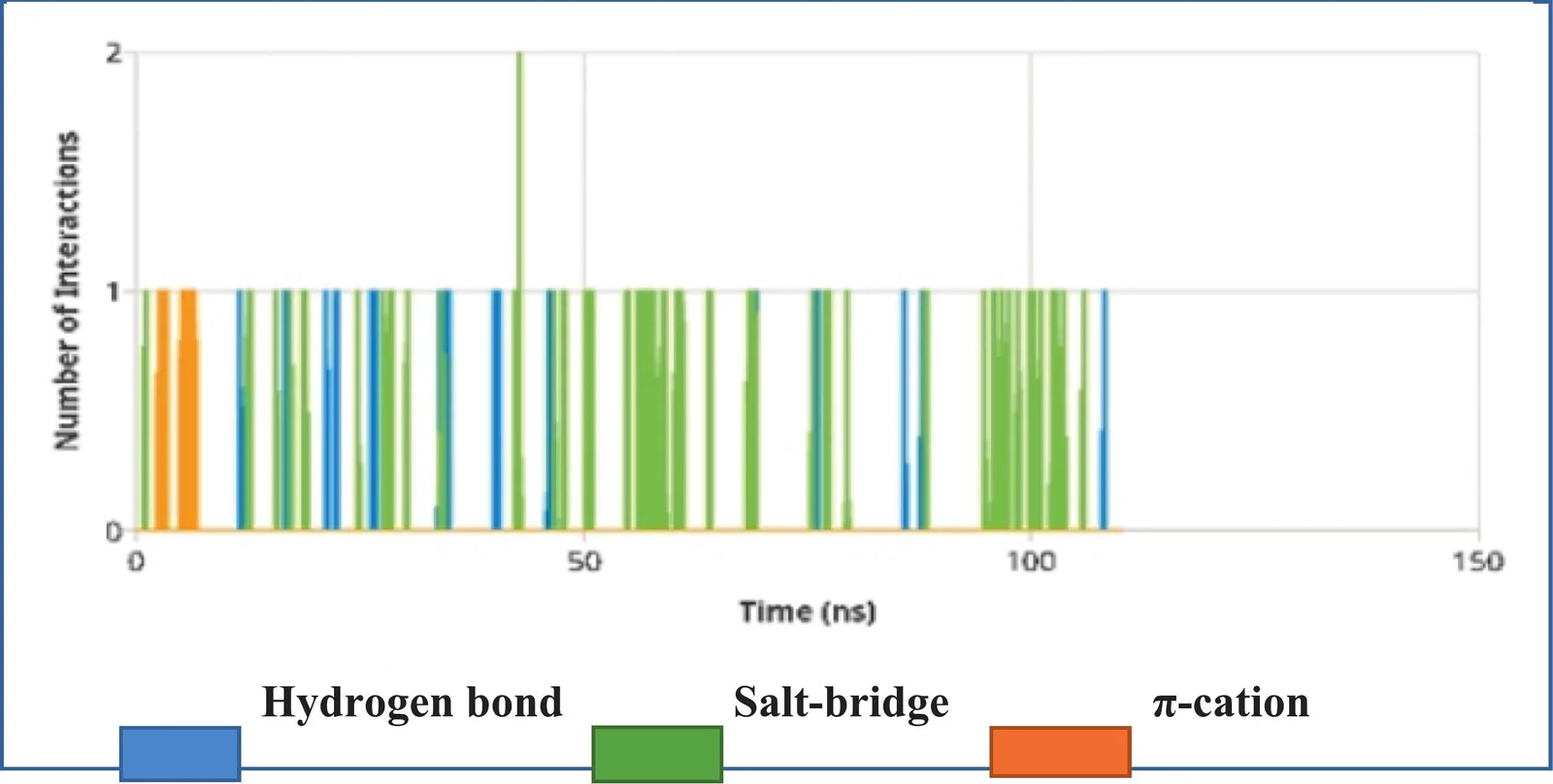
Time-dependent interaction fraction and occurrence graph tracking the continuity of non-covalent interaction (hydrogen bond, salt bridge, and π-cation) between the ligand and the target protein 4HFZ across the 110 ns MD trajectory.

#### 3.6.3 Local binding –site residue dynamics

To evaluate the local flexibility and experimental mobility within the ligand-binding region of the 4HFZ protein, a localized Root Mean Square Fluctuation (RMSF) analysis was conducted alongside its corresponding B-factor profile. As illustrated in Fig. 6, the evaluation focuses on a specific segment of key contact residues on chain A (represented by indices 0 to 8 on the horizontal axis), rather than on the entire protein backbone.

**Fig. 6:**
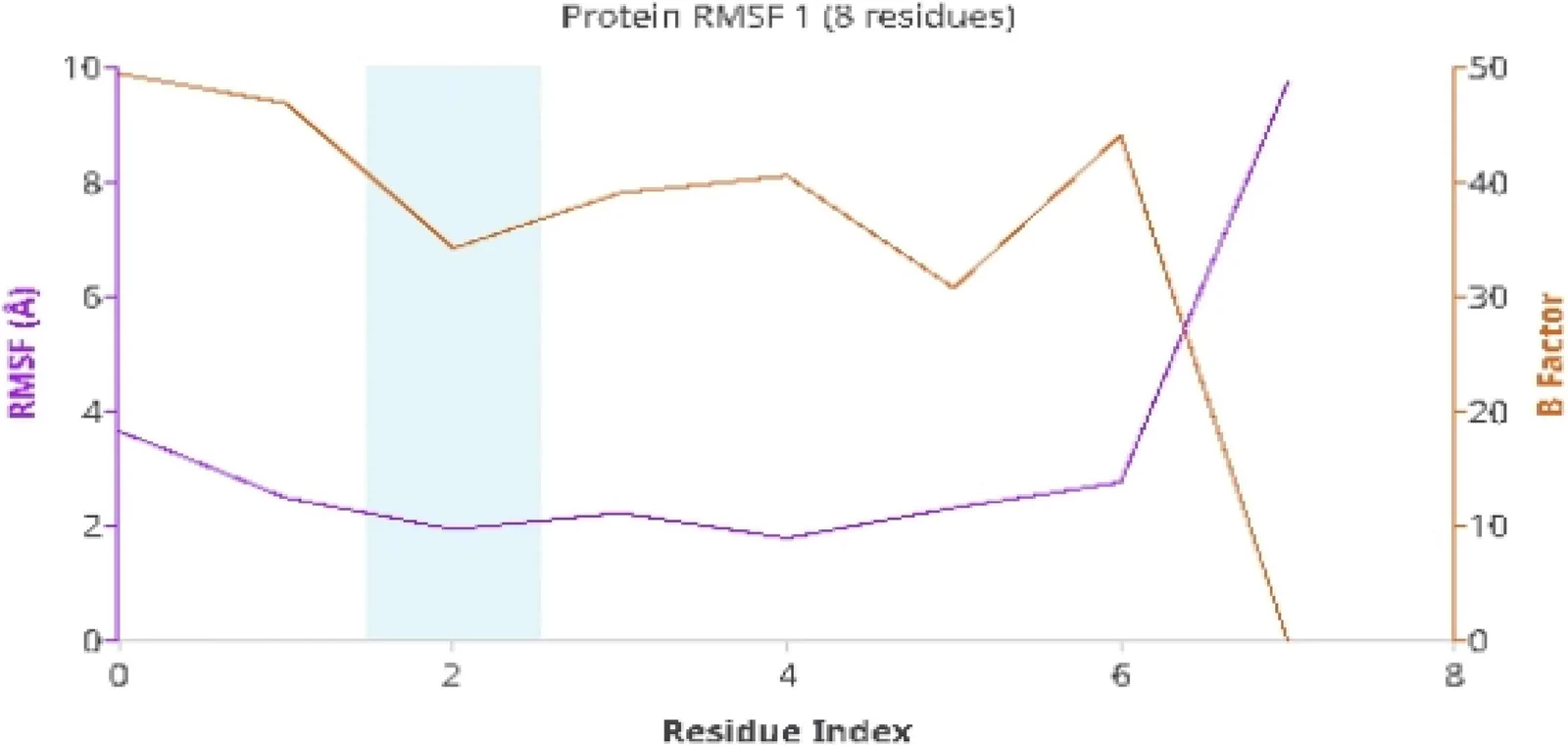
Localized RMSF (Purple line, left axis) and B-factor (orange line, right axis) profiles for the 8 key binding site residues of the 4HFZ protein. The shaded area emphasizes the highly stabilized region at residue index 2. (For interpretation of the references to colour in this figure legend, the reader is referred to the web version of this article.)

The localized RMSF data revealed that each residue within this binding region exhibits a distinct pattern of modification during the molecular dynamics simulation. Certain residues demonstrated considerable flexibility, whereas others maintained relative stability. Specifically, residue THR_26 (Corresponding to index 0) exhibited the highest local fluctuation, with an RMSF value of approximately 3.64 Å, followed by LEU_27 (Index 1), which displayed a moderate degree of flexibility at roughly 2.5 Å.

Conversely, a remarkable conformational rigidity was observed at Residue VAL28 (highlighted within the blue-shaded region at index 2), which exhibited a low RMSF of 1.93 Å. Crucially, this localized stabilization is strongly supported by the experimental thermal factor: the calculated B-factor curve displays a synchronized downward trend, reaching a local minimum of nearly 33 at the same residue index. Similarly, Arg29 (Index 3) maintained comparative stability with an RMSF value of 2.2 Å.

From a structural perspective, these rigid residues (such as Val28) are frequently embedded within well-defined secondary structural elements, such as α-helices or β-sheets, or they undergo tight stabilization due to direct, favorable interactions with the bound ligand within the pocket. In contrast, the higher fluctuations observed in residues such as THR 26 are characteristic of flexible loop regions or terminal edges that accommodate the structural adjustments required for ligand fitting.

Note: In accordance with the localized nature of this analysis, these results are strictly designated to monitor the local dynamic behavior of ligand–contact residues and their contribution to ligand–protein interaction stability, without drawing broader conclusions regarding the global backbone flexibility of the entire 4HFZ protein.

#### 3.6.4 The radius of gyration (*Rg*)

The Radius of Gyration (*Rg*) values ranged between 6.0 Å to 13.9 Å across the 110 ns simulation trajectory (Fig. 7). During the intial relaxation and equilibration phase (0–15 ns), *Rg* exhibited pronounced fluctuation (ranging from ~6.0 Å to 13.9 Å), reflecting substantial initial structural adjustment of the complex in the explicit solvent environment. (See Fig. 8.)

**Fig. 7:**
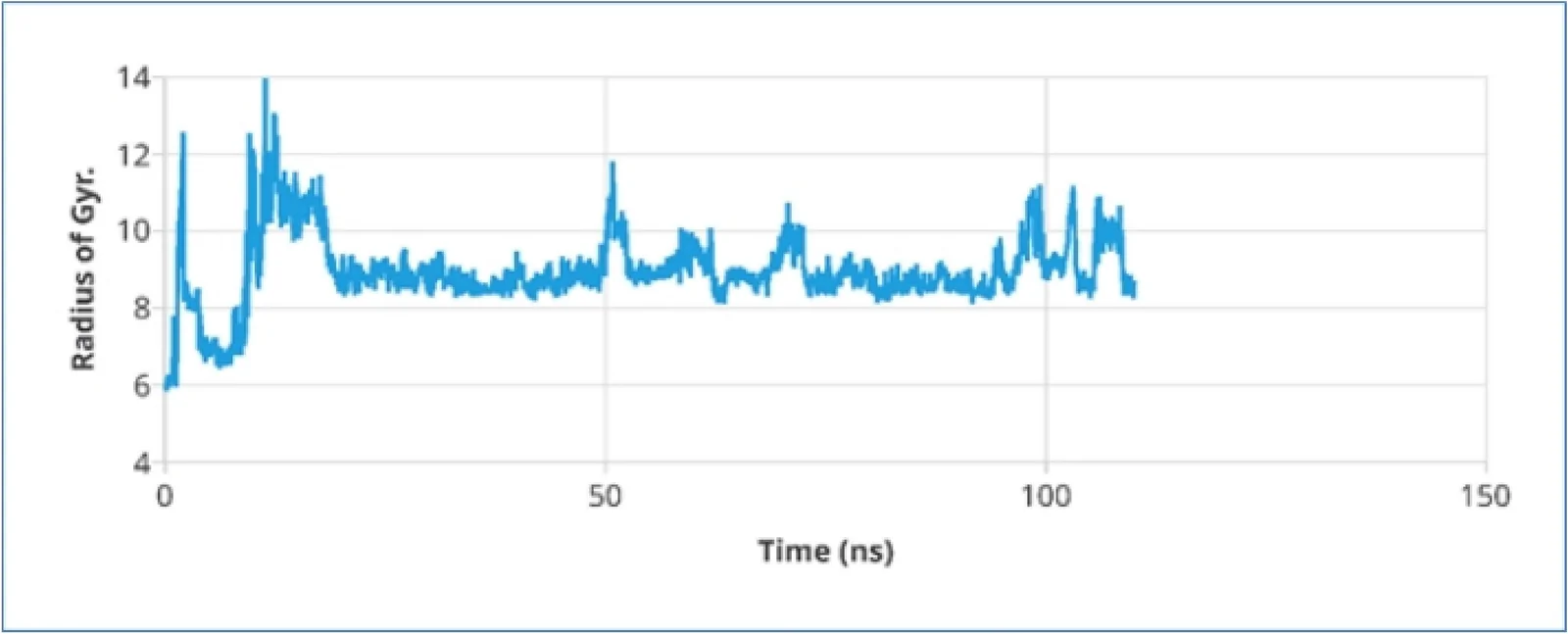
Depicts the radius of gyration (*Rg*) over 110 ns, which demonstrates how tightly the protein-ligand complex is packed. After some changes in the first 10 ns, the *Rg* remains between 8 and 10 Å, indicating that the system remains structurally stable and does not unfold much during the simulation.

**Fig. 8:**
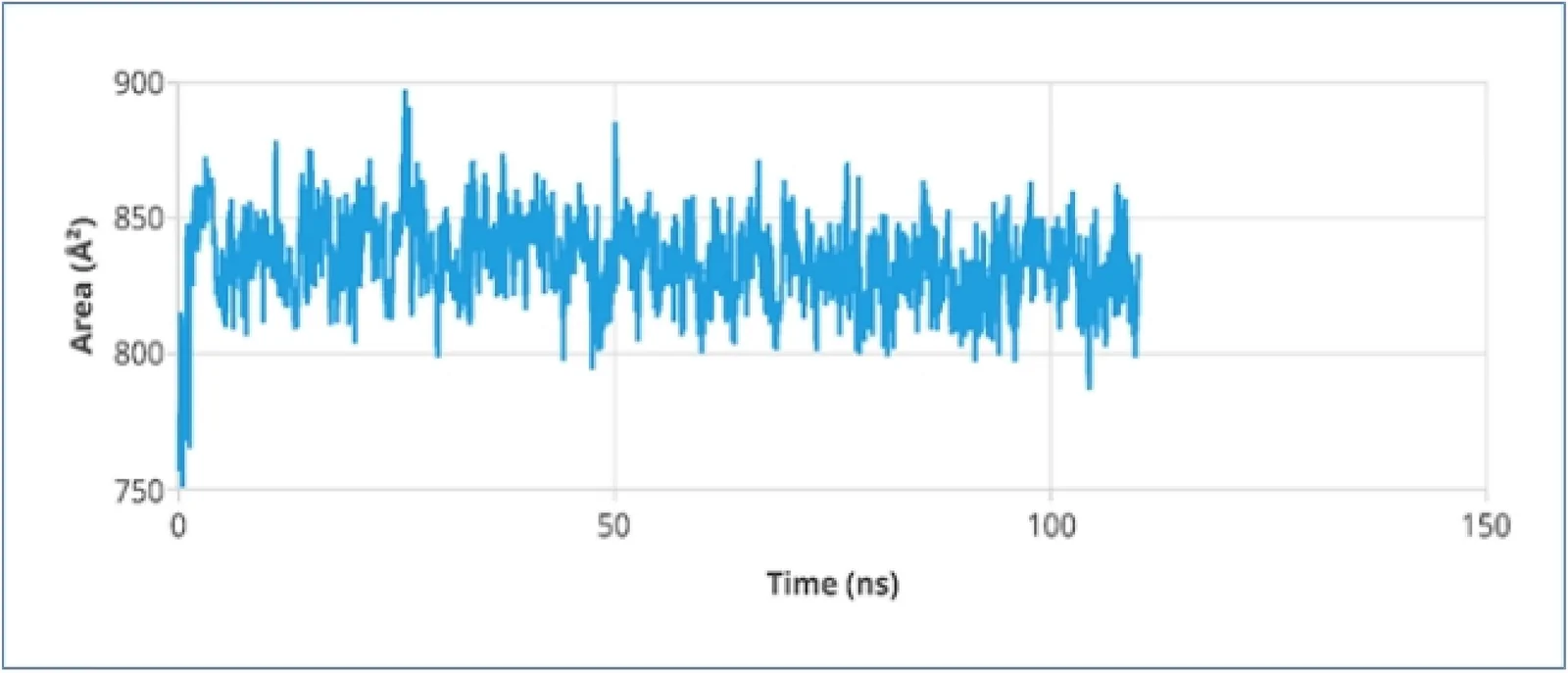
shows how the protein-ligand complex's molecular surface area changes during the course of a 110-ns molecular dynamics simulation. The x-axis shows the simulation time in nanoseconds (ns), while the y-axis shows the molecule surface area in square angstroms (Å^2^). At first, the surface area changes a lot. As the simulation progresses, the data remain stable between about 800 and 860 Å^2^.

Following this initial phase, the complex stabilized remarkably. From 15 ns to 110 ns, the *Rg* values remained within a narrow and consistent range between 8.0 Å and 10 Å (8.83 Å at 28 ns, 9.25 Å at58 ns, and 8.55 Å at 93 ns). This steady trajectory during the production phase confirms that the protein -–ligand complex maintained a compact, stable, and well packed overall structure without undergoing continuous expansion or denaturation.

#### 3.6.5 Molecular surface area analysis

The molecular surface area values observed in the 110 ns simulation range from approximately 768.24 Å^2^ to nearly 863.00 Å^2^. The surface area changes more quickly at the start of the simulation. The molecular surface area tends to stabilize within a narrower range as the simulation progresses. This is especially true between 60 ns and 110 ns, where the surface area stabilizes within a narrower range of ~800 to 860 Å^2^, within the overall trajectory limits (~768 to 863Å^2^).

#### 3.6.6 Polar surface area (PSA)

Polar Surface Area (PSA) values observed over the 110 ns simulation exhibit a relatively steady profile with only minor fluctuations throughout the trajectory. Overall, the PSA values fluctuate within a range of 440 to 510 Å^2^, with core equilibrium values typically remaining between 450 and 488 Å^2^ (averaging ~466 Å^2^). The absence of significant deviations in either direction indicates that no major structural alterations or excessive exposure of previously hidden polar surface areas occurred, confirming the overall conformational stability of the protein–ligand complex (Fig. 9). (See Fig. 10.)

**Fig. 9:**
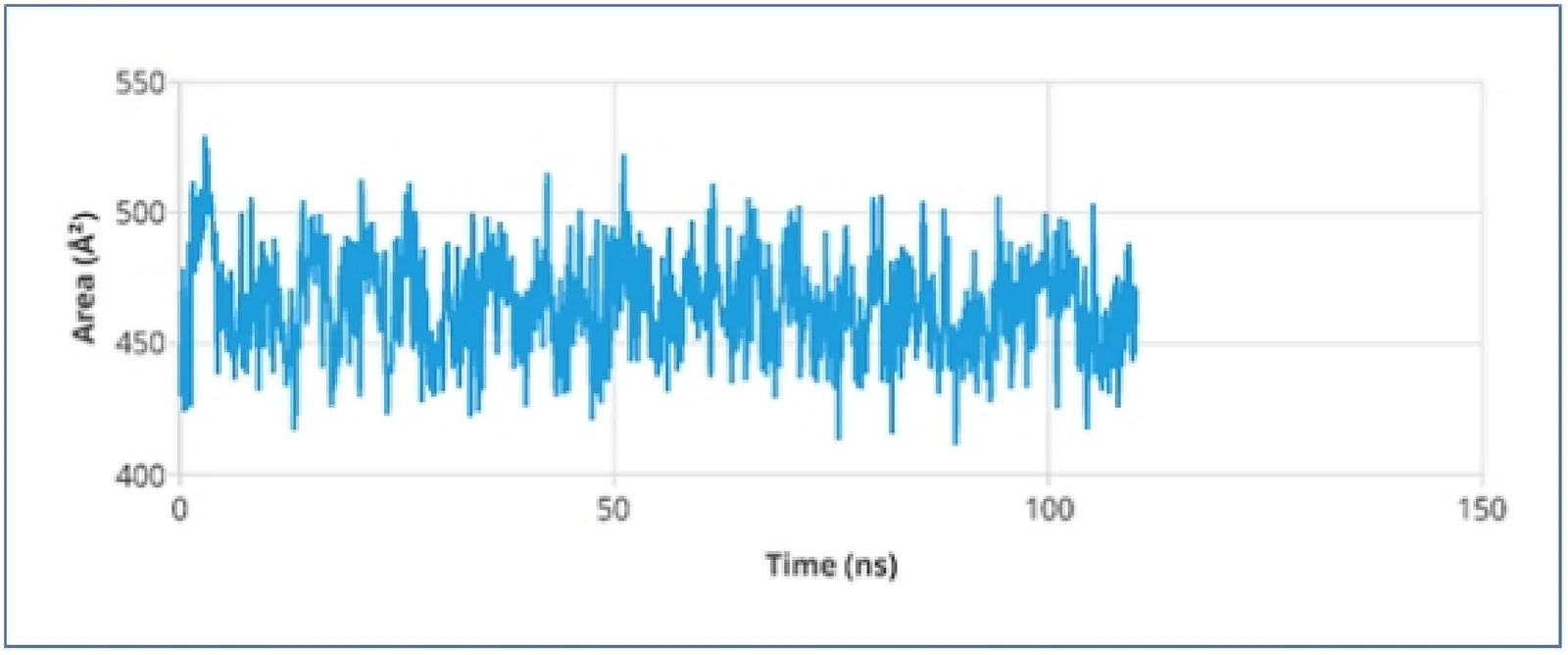
shows the Polar Surface Area (PSA) of the protein–ligand complex. The x-axis shows the time of the simulation in nanoseconds (ns), while the y-axis shows the PSA values in square angstroms (Å^2^). The values fluctuate within an overall range of 440 to 510 Å^2^, with a typical core equilibrium between 450 and 488 Å^2^.

**Fig. 10:**
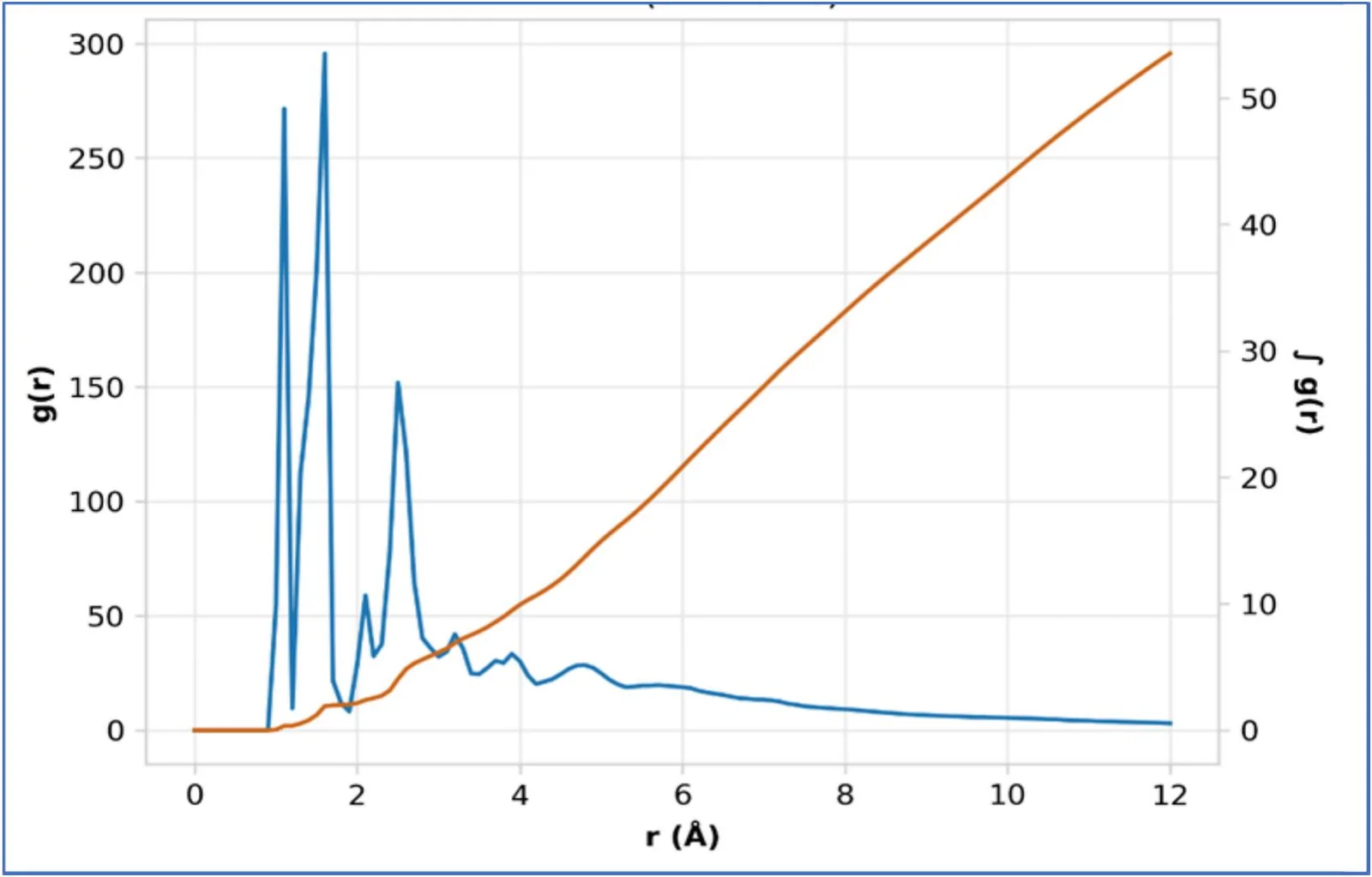
Displays the Radial Distribution Function (RDF). The blue line, denoted as *g*(r), shows the probability of finding a particle at a specific distance. The x-axis, labeled r, represents the radial distance. (For interpretation of the references to colour in this figure legend, the reader is referred to the web version of this article.)

#### 3.6.7 Radial distribution function (RDF)

The radial distribution function (RDF) represents how atoms are distributed around a reference atom within a molecular system over the course of the simulation. The x-axis (r, in Å) shows the radial distance, while the blue line *(g*(r)) indicates the probability of finding a particle at that distance. The orange line (∫*g*(r)) is the integration of the RDF, showing the cumulative number of neighboring atoms up to a given distance. The sharp peaks at short distances (1–3 Å) indicate strong, structured interactions such as hydrogen bonding or close contact between ligand and surrounding residues, suggesting a well-defined local structure**.**

## 4 Discussion

The study revealed that, in terms of demographic characteristics, there was no significant difference in age and body mass index (BMI) between smokers and controls. It suggests that these demographic factors are unlikely to bias study outcomes. Notably, the observed high levels of p53 protein in smokers serve as a physiological indicator of cellular defense mechanisms attempting to counteract severe DNA damage, reinforcing the concept that TP53 expression acts as a marker of exposure rather than a direct oncogenic driver.^24^

This response is mediated by upstream kinases such as ATM/ATR, which phosphorylate p53, thereby disrupting its MDM2-mediated degradation, stabilizing the protein and facilitating cell cycle arrest or apoptosis. Crucially, our analysis demonstrated highly significant differences in cumulative smoking exposure, measured in pack-years, across the stratified cohorts (29.94 ± 13.46 for cigarettes, 14.90 ± 6.55 for vape, and 11.07 ± 9.15 for narghile). This pronounced divergence confirms that each consumption modality imposes a distinctly different quantitative and chemical baseline burden of xenobiotic exposure on the cellular environment. Intriguingly, despite these significant differences in exposure intensity, the continuous subgroup correlation analysis between pack-years and serum p53 levels did not yield a linear, statistically significant correlation among conventional cigarette smokers (*r* = 0.010, *p* = 0.929) or narghile users (*r* = −0.019, *p* = 0.910). Rather than undermining the toxicological impact of combustible tobacco, this discordance between distinct group means and a lack of continuous linear correlation strongly supports a ‘biphase response’ or a strict ‘threshold model.‘.

Upon initiating combustible tobacco use, the rapid influx of combustion-derived polycyclic aromatic hydrocarbons (PAHs), such as Benzo[*a*]pyrene, rapidly triggers the cellular emergency pathway, maximizing p53 levels to repair bulky DNA adducts at guanine bases or to prompt apoptosis.^25^ Once this maximal protective threshold is reached, further cumulative exposure over higher pack-years does not linearly drive serum protein expression upward; instead, the pathway enters a saturated plateau phase where serum levels decouple from a simple linear dose-response curve, eventually transitioning into a phase of localized genomic exhaustion where fixed mutational signatures predominantly Guanine: Cytosine to Thymine: Adenine (G T) transversions at hot-spot codons, take place.^26^

Similarly, while narghile smoke shares this PAH-driven genotoxic pathway due to tobacco combustion, its unique reliance on burning charcoal introduces disproportionately higher levels of carbon monoxide and heavy metals, such as lead, chromium, and nickel.^27^ Even with a lower cumulative pack-year profile (11.07 ± 9.15), these heavy metals synergistically amplify the generation of reactive oxygen species (ROS), inducing pervasive indirect oxidative DNA lesions—such as 8-hydroxy-2′-deoxyguanosine (8-OHdG), which forces a distinct compensatory pattern in serum P53 levels to counter systemic oxidative stress rather than driving a linear correlation.^28^

Conversely, a distinctly non-canonical and alarming trend was captured within the vaping cohort. Despite demonstrating intermediate pack-year exposure (14.90 ± 6.55), vape users exhibited a notable, moderate negative correlation (*r* = −0.418, *p* = 0.075) between cumulative exposure and serum p53, closely approaching statistical significance. This inverse trend warrants a specialized toxicological explanation. Unlike combustible tobacco, vaping introduces systemic exposures to thermal degradation products of the liquid vehicles (propylene glycol and vegetable glycerin)—predominantly reactive aldehydes such as formaldehyde, acetaldehyde, and acrolein—alongside metallic nanoparticles (nickel, copper) leached from heating coils.^29^ While initial vaping exposure provokes an early spike in p53 to counter aldehyde-induced DNA-protein crosslinks (DPCs).^30^ prolonged and higher cumulative exposure induces direct proteotoxic stress.^31^

The complex undergoes initial structural adjustments and minor fluctuations, eventually settling into a stable *Rg* plateau starting from ~15 Specifically, heavy metals such as nickel can directly bind to and disrupt the critical zinc-finger domain of the p53 protein, which reflects circulating protein concentrations rather than direct functional dynamics within cells. Consequently, concepts such as potential threshold behavior or changes in MDM2–p53(4HFZ) binding should be interpreted with caution as hypothesis-generating observations that require further functional validation in the laboratory.^32^ This provides a plausible biological rationale for why prolonged, cumulative vaping exposure, unlike traditional smoking, trends toward a progressive reduction in detectable serum p53, highlighting a fundamental mechanistic divergence where e-cigarette aerosols progressively suppress or exhaust the functional integrity of the p53 pathway over time.”

Despite these highly varied toxic inputs, ranging from combustible polycyclic aromatic hydrocarbons and heavy metals in tobacco smoke to thermally decomposed aldehydes **(**acrolein, formaldehyde) in e-cigarette vapor, no statistically significant difference was observed in serum p53 protein concentrations among the three smoker subgroups (*p* = 0.547). This statistical equivalence strongly suggests that, regardless of the specific chemical composition of the foreign substances, each delivery mechanism induces sufficient intracellular oxidative stress, generating reactive oxygen species and subsequent toxic gene damage to fully activate and saturate the systemic p53 defense loop. Consequently, despite the different chemical triggers, they all lead to the same clinical phenotype of p53 activation.^33^

Computational molecular docking indicates that nicotine binds to the MDM2-P53 complex (4HFZ) with moderate affinity, with a predicted binding energy of −5.31 kcal/mol. The predicted binding affinity of nicotine (−5.31 kcal/mol) indicates a moderate binding affinity for the MDM2-p53 complex, in contrast to synthetic small-molecule inhibitors with high affinity. Furthermore, the stabilization of the RMSD trajectory at around 1.94 Å reflects conformational adjustments and structural flexibility inherent in the compound's outer rings in an open solvent environment, rather than structural collapse. However, the lack of parallel molecular dynamics simulations of the protein in its free state limits the ability to isolate nicotine's specific stabilizing effects.^34^

A lower RMSD usually indicates a more reliable docking result, as the anticipated pose is closer to an ideal or empirically validated orientation. Analyzing the components of the docking score reveals the reasons for pose1 superiority.^35^, ^36^

The E_conf (22.62) shows the internal energy of the ligand conformation. This number is not very high, indicating that the ligand is not under much stress and remains in a relatively stable conformation. The E_place score (−6.07) shows how well the ligand fits into the binding pocket during the placement stage. A larger negative value means that the ligand and the pocket are better at complementing each other's shapes and interactions. The main thing that Val28 does is build a hydrogen bond with nicotine, which makes the complex more specific and stable. Arg29 is very important because it interacts with nicotine in many ways. It forms two hydrogen bonds with nicotine's nitrogen atoms and a π-cation interaction between Arg29 guanidinium group and nicotine's aromatic ring. This makes the molecule even more stable by drawing it in through electrostatic attraction.^37^, ^38^

As evidenced by the RMSD profile (Fig. 4), which exhibited an initial sharp deviation approaching 15 Å before establishing a highly steady plateau around 8 Å for the remainder of the 110 ns simulation. This temporary early spike reflects a critical structural relaxation and solvent equilibration phase, during which the protein-ligand complex adjusted its geometry from its static, docking-derived pose to a dynamically optimized conformation under explicit solvent conditions.^39^ Crucially, the absence of a continuous, progressive upward drift throughout the production phase strictly rules out global or partial protein unfolding, confirming that the overall fold remained fully preserved.^40^

This dynamic adjustment ultimately validates the docking affinity, ensuring the sustained structural integrity of the complex and safeguarding the key interaction with Arg29 and Val28 initially identified in the static docking pose. Such behavioral profiling underscores the need to evaluate geometric descriptors, such as RMSD, alongside interaction persistence.

This comprehensive approach aligns with recent investigations into lung cancer targets, in which combining MD trajectories with robust molecular fingerprinting provides a reliable framework for evaluating conformational stability and distinguishing transient equilibration–related adjustments from true structural destabilization.^41^

The Analysis of the 110 ns MD simulation reveals a dynamic interplay of non-covalent interactions between nicotine and the 4HFZ protein. While the initial docking pose suggested a dominant role for a π-cation and π-π stacking interaction, their presence was transient and primarily restricted to the early stages of the simulation (≤ 11.0 ns). subsequently, the complex's stability was predominantly maintained by persistent hydrogen bonds and strong salt bridges (peaking at an interaction fraction of 0.20), reflecting a transition from short- lived, aromatic interactions toward a more dynamically sustained binding network.^42^ This underscores the necessity of considering structural plasticity and conformational dynamism in protein ligand systems, as static docking predictions often fail to reflect long-term stability. As visually demonstrated in the continuous interaction profile (Fig. 5), the complex undergoes time- dependent conformational adjustments, capturing the persistence of these non-covalent network across the 110 ns trajectory, while Table 6 provides the quantitative interaction fraction for each discrete time interval. This shift toward persistent salt bridge and hydrogen bonds indicates a developing stabilization mechanism, a common phenomenon observed in MD trajectories. Further free energy calculation (e.g., MM/PBSA) are recommended to quantitatively evaluate the energetic contribution of the transient pi-interaction loss relative to the overall binding affinity.^43^

The localized dynamic behavior of the 4HFZ binding pocket provides critical insights into the structural and thermodynamic stabilization of the Nicotine-4HFZ complex. The clear gradient of flexibility observed within this 8-residue segment highlights a functional dichotomy between different regions of the binding site. The elevated mobility at the terminal edges, specifically Thr_26 and Leu_27, is highly characteristic of flexible loop conformations. Structurally, this localized plasticity is essential, as it provides the protein with the conformational freedom needed to undergo induced-fit adjustments, thereby facilitating the optimal entry and positioning of Nicotine.^44^

Conversely, the remarkable rigidity observed at the core of the pocket, exemplified by the minimal RMSF values and the synchronized experimental B-factors for Val28 (at residue index 2) and Arg29, indicates a highly optimized anchoring mechanism. By maintaining structural stability throughout the 110 ns simulation, these key contact residues preserve the spatial geometry required to sustain critical non-covalent interactions.^45^ This local stabilization is instrumental in preventing premature ligand dissociation, thereby ensuring the persistence of essential hydrogen bonds and salt bridges that govern overall complex stability. The alignment between the local RMSF profiles (purple line) and their corresponding B-factors reinforces the conformational stability of the active site, validating that computational fluctuations match experimental thermal mobility.^46^

To contextualize these findings and address potential interpretative boundaries, it must be emphasized that this analysis is strictly limited to the localized binding interface, not to the global protein architecture. Ideally, a comprehensive whole-backbone RMSF profile would complement these observations by mapping global macromolecular flexibility. However, due to the unavailability of the complete simulation trajectory files during the revision stage, a full-backbone recalculation could not be realized. Acknowledging this limitation, the study deliberately refrains from extrapolating these localized fluctuations to represent the global backbone behavior of the 4HFZ protein as a whole. Instead, this targeted approach serves as a high-resolution, focused descriptive lens that isolates the specific active-site dynamics directly impacting ligand retention, providing a scientifically rigorous assessment of interface stability without introducing overinterpretations.^47^

The Radius of Gyration (*Rg*) was analyzed throughout the 110 ns molecular dynamics simulation to quantify the structural compactness and overall stability of the protein–ligand system. During the first 15 ns, the complex undergoes initial structural adjustments and minor fluctuations, eventually settling into a stable *Rg* plateau starting from ~15 ns through the remainder of the 110 ns simulation. While tracking the *Rg* profile is crucial for monitoring structural integrity and behavior upon ligand binding, it is important to acknowledge that a comparative baseline simulation for the unbound Apo-protein (4HFZ without nicotine) was not performed. Consequently, these findings are interpreted as characterizing the structural behavior and sustained compactness of the nicotine-bound complex itself, rather than as definitive proof of ligand-induced stabilization relative to the unliganded state.^48^, ^49^ Distinguishing nicotine-specific conformational alterations from the protein's intrinsic dynamics would require an independent Apo-control simulation, which remains a limitation of the current setup.

The evolution of the Molecular Surface Area (MSA) during the 110 ns MD simulation indicates that the protein-ligand complex rapidly approaches a dynamic equilibrium. Overall, the surface area fluctuates within the range of 768.24 Å^2^ to 863 Å^2^, maintaining a stable equilibrium range of ~830–850 Å^2^ throughout the trajectory. At the same time, the sustained fluctuations observed throughout the remainder of the trajectory reflect the complex's intrinsic structural flexibility in an aqueous environment. However, because no independent simulation of the unliganded Apo-protein was conducted as a baseline control, these surface configurations and polar surface area (PSA) properties cannot be unequivocally attributed to nicotine-specific shielding or surface stabilization. Instead, they describe the baseline topology and solvent-exposure dynamics of the complexed state.^50^, ^51^

Despite the limitations associated with the absence of an Apo-protein control, advanced computational docking and atomistic MD simulations remain indispensable tools for mapping intricate bimolecular interactions and regulatory cascades. This in silico framework is especially valuable when exploring the multi-target (polypharmacological) modulation of cancer-associated pathways, such as those governing the p53 regulatory network. For instance, Ahmad and Raza (2025) successfully deployed an advanced computational workflow using molecular docking. They extended MD simulations to evaluate the polypharmacological potential of Imidurea against key lung cancer-associated proteins, including EGFR, CDK2, STAT3, BCL2, p53, and ABCB1. Their insights demonstrate how detailed interaction profiling and structural trajectories can decode complex ligand modulation within oncogenic systems. Aligning with this paradigm, our simulation of the nicotine–4HFZ system offers preliminary insights into the structural dynamics of this complex system, reinforcing the utility of computational pipelines for characterizing p53-associated macromolecular targets.^52^

Furthermore, the radial distribution function (RDF) characteristic the local structural ordering and solvation dynamics of the nicotine–4HFZ complex within the explicit TIP3P water environment. The RDF profile, *g*(r), exhibits distinct sharp peaks at short radial distances (< 2.5 Å), confirming strong hydrogen bonding and favorable polar coordination shells between the protein–ligand complex and surrounding water molecules. The rapid dampening of *g*(r) toward 1 at larger distances indicates the absence of long-range crystalline order, fully consistent with an explicit-solvent atomistic MD simulation of a macromolecular system.^53^

### 4.1 Limitations of the study

Several limitations of this study should be acknowledged. Firstly, the serum ELISA assay measures circulating levels of the p53 protein, which do not directly reflect intracellular p53 signaling or functional changes. Secondly, the pack-year equivalents calculated for exposure to shisha and e-cigarettes are approximate mathematical estimates that lack global standardization. Thirdly, our computational model lacked a baseline for simulating unbound protein, and the binding rate obtained was moderate.

## 5 Conclusion

This study demonstrates that smoking is associated with a notable rise in serum P53 concentration, reflecting increased cellular stress and potential DNA damage among smokers. Furthermore, molecular docking and molecular dynamics simulations suggest that nicotine can interact effectively with the MDM2-P53 complex, particularly through key contacts with VAL28 and Arg29. While these computational findings suggest a possible molecular interaction within the MDM2-P53 regulatory axis, they remain hypothesis-generating. They cannot definitively establish a direct functional or clinical outcome regarding cancer risk without further experimental validation. Notably, the lack of a baseline comparison with the unbound Apo-protein (4HFZ) limits the ability to conclusively determine whether the observed polar surface area (PSA) and Radius of Gyration *(Rg*) profiles are specifically induced or stabilized by nicotine. Advanced computational docking and molecular simulation provide an essential framework for further elucidating the polypharmacological modulation of p53 and its associated regulatory proteins. Ultimately, to bridge the gap between these in silico observations and biological reality, future in vitro studies, including MTT-based cell viability assays, apoptosis analysis, and expression profiling, are warranted to establish the definitive pathological or therapeutic significance of nicotine's effect on p53 activation.

## CRediT authorship contribution statement

**Noor Kareem Khalifa:** Writing – review & editing, Writing – original draft, Software, Methodology, Investigation, Formal analysis, Conceptualization. **Maysoon Khalid Hussein:** Writing – review & editing, Writing – original draft, Visualization, Validation, Supervision, Methodology, Conceptualization.

## Ethics declaration

Written informed consent to take part in the study and to publish the article has been obtained from all participants or their legal representatives. The privacy rights of participants have been observed.

This study was performed in compliance with relevant laws, regulatory frameworks and guidelines where the research took place. This study was approved by the Ethics Committee of the College of Science, University of Baghdad. (Approval No. Ref. CSEC/0325/0054)

This research follows the CARE guidelines and the CARE checklist. Ethics approval: Ref:CSEC/0325/0054. Date:18/2/2026.
